# Neurofilaments in progressive multiple sclerosis: a systematic review

**DOI:** 10.1007/s00415-020-09917-x

**Published:** 2020-05-23

**Authors:** Thomas Williams, Henrik Zetterberg, Jeremy Chataway

**Affiliations:** 1 Department of Neuroinflammation, Faculty of Brain Sciences, Queen Square Multiple Sclerosis Centre, UCL Queen Square Institute of Neurology, University College London, London, UK; 2 Department of Neurodegenerative Disease, Faculty of Brain Sciences, UCL Queen Square Institute of Neurology, University College London, London, UK; 3 UK Dementia Research Institute, University College London, London, UK; 4 Department of Psychiatry and Neurochemistry, Institute of Neuroscience and Physiology, The Sahlgrenska Academy At the University of Gothenburg, Mölndal, Sweden; 5 Clinical Neurochemistry Laboratory, Sahlgrenska University Hospital, Mölndal, Sweden; 6 Biomedical Research Centre, National Institute for Health Research, University College London Hospitals, London, UK

**Keywords:** Progressive multiple sclerosis, Multiple sclerosis, Biomarkers, Neurofilament light chain (NFL), Neurofilament heavy chain (NFH)

## Abstract

**Background:**

Neurofilament proteins have been extensively studied in relapsing–remitting multiple sclerosis, where they are promising biomarkers of disease activity and treatment response. Their role in progressive multiple sclerosis, where there is a particularly urgent need for improved biomarkers, is less clear. The objectives of this systematic review are to summarise the literature on neurofilament light and heavy in progressive multiple sclerosis, addressing key questions.

**Methods:**

A systematic search of PubMed, Embase, Web of Science and Scopus identified 355 potential sources. 76 relevant sources were qualitatively reviewed using QUADAS-2 criteria, and 17 were identified as at low risk of bias. We summarise the findings from all relevant sources, and separately from the 17 high-quality studies.

**Results:**

Differences in neurofilament light between relapsing–remitting and progressive multiple sclerosis appear to be explained by differences in covariates. Neurofilament light is consistently associated with current inflammatory activity and future brain atrophy in progressive multiple sclerosis, and is consistently shown to be a marker of treatment response with immunosuppressive disease-modifying therapies. Associations with current or future disability are inconsistent, and there is no evidence of NFL being a responsive marker of purportedly neuroprotective treatments. Evidence on neurofilament heavy is more limited and inconsistent.

**Conclusions:**

Neurofilament light has shown consistent utility as a biomarker of neuroinflammation, future brain atrophy and immunosuppressive treatment response at a *group* level. Neither neurofilament light or heavy has shown a consistent treatment response to neuroprotective disease-modifying therapies, which will require further data from successful randomised controlled trials.

**Electronic supplementary material:**

The online version of this article (10.1007/s00415-020-09917-x) contains supplementary material, which is available to authorized users.

## Introduction

Progressive multiple sclerosis (PMS) is characterised by a steady accumulation of disability largely independent of relapses [1]. In primary progressive multiple sclerosis (PPMS), progression occurs from onset without preceding relapses; in secondary progressive multiple sclerosis (SPMS), progression follows an initial relapsing and remitting phase of the disease. In both cases, progression may occur either in association with inflammatory activity (active progression), or in the absence of such inflammatory activity (non-active progression) [1].

In contrast to relapsing–remitting multiple sclerosis (RRMS), where there has been a rapid expansion in available treatments, few treatments are available for PMS, and these are restricted to those with active progression. There is a clear need to innovate the therapeutic pipeline, particularly in non-active PMS, to enhance the development of novel treatments. This ideally would involve moving beyond MRI-based biomarkers of treatment efficacy in clinical trials. A lead candidate for this is neurofilament light (NFL), and to a lesser extent neurofilament heavy (NFH), cytoskeletal proteins released from neurones following injury. With the advent of highly sensitive digital enzyme-linked immunoassay (ELISA), also called Single molecule array (Simoa), platforms, neurofilaments can now be sensitively quantified in both cerebrospinal fluid (CSF) and blood, making application to large cohorts and clinical trials a practical reality [2].

In RRMS, neurofilaments are associated with clinical and MRI inflammatory activity, and predict future disability progression [3–5]. Their ability to demonstrate treatment response has led to the suggestion that NFL may replace established MRI-based outcomes in phase 2 trials in RRMS [6]. Similarly, if issues around assay standardisation and inter-laboratory precision can be resolved, and larger datasets on normative values established, serial bNFL monitoring is likely to become part of standardised disease activity monitoring in RRMS in the near future [7, 8]. RRMS studies will not be considered further in this review.

The place of neurofilaments in PMS, however, is less well studied, and the underlying association with inflammatory activity questions their utility in non-active PMS.

The aims of this systematic review are to qualitatively summarise the literature on the role of neurofilaments (NFL and NFH, CSF and blood) in PMS. Specific questions include:

1. In patients with PMS, are neurofilament concentrations in CSF or blood associated with current disease course and cross-sectional measures of inflammatory activity and disability?
2. In patients with PMS, are neurofilament concentrations in CSF or blood associated with future measures of disability progression?
3. In patients with PMS, are neurofilament concentrations responsive markers of disease-modifying treatment (DMT) in observational or randomised controlled trials?

## Methods

Our systematic review was guided by PRISMA [9]. Any original study reporting neurofilament data in patients with PMS was identified. We included published research papers, conference abstracts and conference presentations, with no restrictions on date or language. In studies including mixed cohorts of patients (RRMS and PMS), data had to be separately presented or described for PMS to be included.

**Fig. 1:**
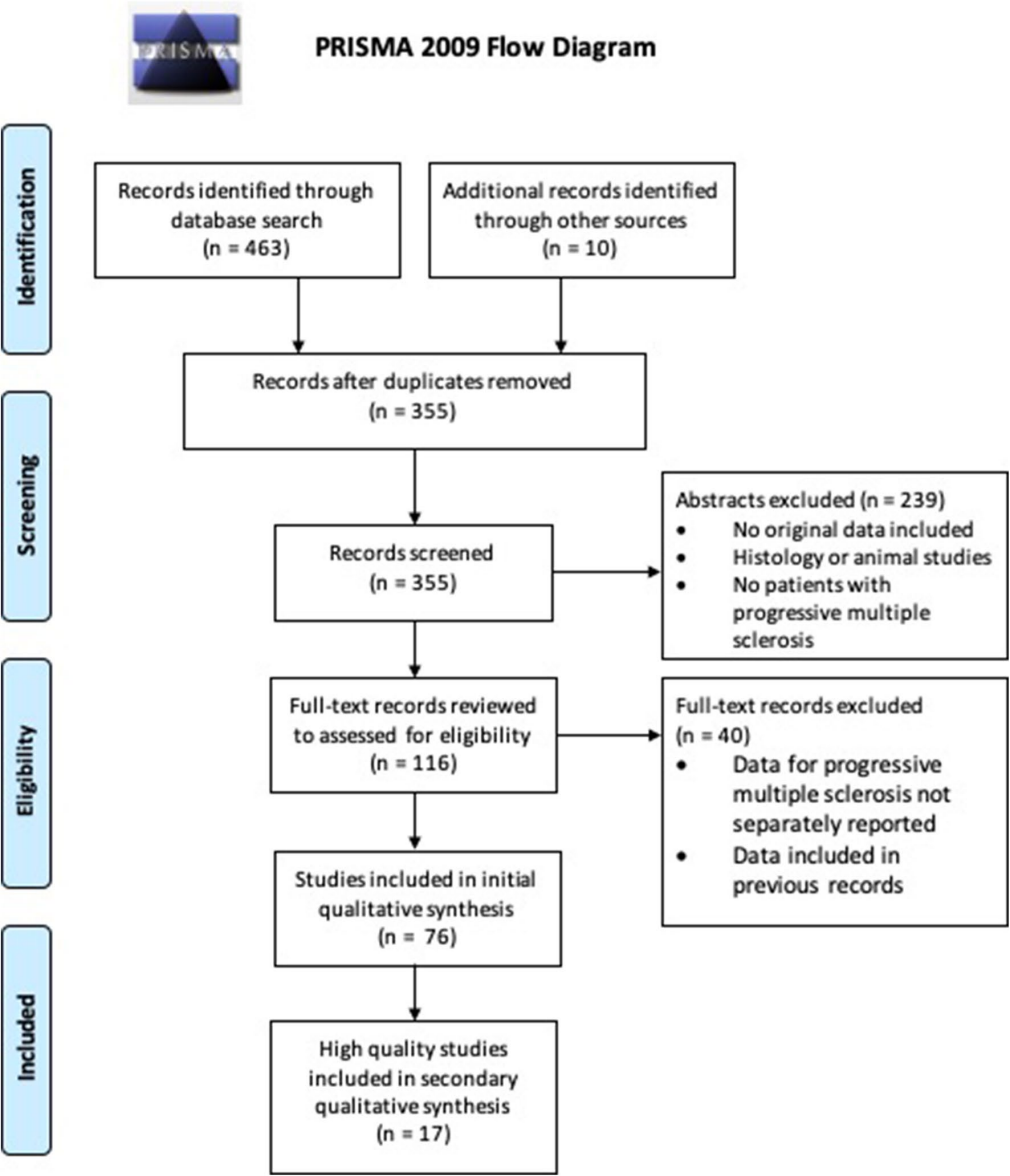
PRISMA Flow Diagram. Due to the limited number of studies at low risk of bias, all literature applicable to each review question is first summarised, followed by a summary of studies at low risk of bias. A quantitative meta-analysis was not undertaken due to heterogeneity in study data and limited data availability

One author developed a searched strategy and interrogated PubMed, Embase, Web of Science and Scopus in December 2019 using the search terms (“neurofilament” OR “neurofilaments”) AND (“progressive” AND “multiple sclerosis”). We identified 463 records (Fig. 1), and a further ten records were identified from a review of online conference libraries and the author’s own records. 118 duplicates were removed and a further 239 records were removed following a review of abstracts. The most common reasons for record exclusion were that no original data were reported, records reported histological or animal data rather than human fluid biomarkers, or that no PMS patients were included in the study. The remaining 116 records were reviewed in full, and a further 40 excluded as they did not separately report neurofilament findings in PMS-only cohorts or contained datasets already included from other records.

The remaining 76 records were qualitatively reviewed. The QUADAS-2 tool was used to assess for risk of bias. For each publication, the reference standard was defined as that to which the neurofilament data was being compared. The PRISMA table and literature review data are available in Online Resources 1 and 2. Due to the limited number of high-quality studies, we first present a qualitative review of all eligible studies regardless of QUADAS scoring, to give the most complete review of the literature. We shall then summarise the 17 high-quality studies separately. Summary statistics were obtained from the published records. A quantitative meta-analysis was not undertaken due to heterogeneity in the data (CSF and blood, variability in assays used to quantify neurofilament), and limits on data availability from conference abstracts and presentations.

## Results

### Neurofilament light

#### NFL—associations with disease course

A number of studies (including 312 PMS patients) have reported that CSF or blood NFL (cNFL/bNFL) is *higher* in PMS compared to RRMS [4, 5, 10–19], or that it increases more quickly in PMS [20]. Others, however, report that NFL is *lower* in PMS compared to RRMS [21–23] or controls [24]. The majority of studies found no significant difference between disease stages [25–38].

Concurrent disease activity significantly impacts comparisons of NFL between disease states. RRMS in remission have similar cNFL to PMS [25, 39, 40], a finding replicated in a recent meta-analysis—a significant difference between RRMS and PMS patients was lost once patients currently experiencing a relapse were excluded [41].

Similarly, two studies have reported associations between bNFL and disease course in univariate analyses, but this significance is lost in multivariate analyses, where significance only persisted for age, EDSS, recent relapses and DMT treatment status [4, 5]. One large study has reported comparisons between bNFL in PMS subtypes, suggesting that bNFL is higher in SPMS compared to PPMS. This persists independent of MRI inflammatory activity [42].

#### NFL and disease activity in PMS

Disease activity was defined as either recent relapses, T1 GAD-enhancing lesions or new/enlarging T2 lesions. Replicating findings in RRMS populations, both cNFL and bNFL have been consistently reported to be higher in PMS patients with disease activity compared to PMS patients without disease activity [3, 41–48]. A minority of studies reported no such associations between c/bNFL and disease activity in PMS [5, 49, 50].

#### NFL and current disability

Measuring disability through EDSS, timed 25-foot walk or nine-hole peg test, both cNFL and bNFL have been associated with current disability in PMS [4, 10, 12, 19, 37, 42, 47]. cNFL has also been associated with cognitive performance [51]. There is, however, heterogeneity in the literature, with a number of studies reporting no such association with cNFL [35, 43, 44, 52–58] or bNFL [58, 59].

#### NFL and cross-sectional MRI biomarkers

Beyond measures of disease activity, NFL has been associated with other MRI biomarkers of MS pathology. cNFL has been reported to be associated with cortical thickness, T1 hypointense lesion volume and magnetisation transfer ratios in normal appearing white matter and grey matter [49, 57, 60]. In larger studies of sNFL, associations have been found with T2 lesion volume [42, 46, 47], and a recent study has found associations with the presence of chronic active lesions. The latter were defined by the presence of paramagnetic rims on T2* sequences, and PMS patients with two or more chronic active lesions had significantly higher sNFL compared to those with 0–1 chronic active lesions [61].

#### NFL and other biomarkers of inflammatory activity or disability

In addition to the associations with clinical and MRI evidence of disease activity, cNFL has been reported in association with other fluid biomarkers of CNS inflammatory activity, including osteopontin, CXCL13, CSF lymphocyte count, CSF IgG index, sCD27 and sCD14 [44, 60, 62]. Both cNFL and bNFL have also been associated with markers of glial pathology, including glial fibrillary acidic protein (GFAP), chitinase-3-like 1 protein (CHI3L1) and soluble triggering receptor expressed on myeloid cells 2 (sTREM2) [35, 37, 44, 45, 58, 62]. A single study found associations between bNFL and ocular coherence tomography (OCT) markers of neurodegeneration, but this was not significant in the PMS-only cohort [63].

#### NFL and future disability

Two studies have reported associations between baseline cNFL and future disability progression in PMS cohorts [54, 64], whilst another found no association [65]. With bNFL, one study reported no association with future disability progression [10], but this is contradicted by three larger studies. In the ORATORIO study of ocrelizumab in PPMS, a tenfold increase in baseline bNFL in the control group was associated with increased risk of progression on 9-hole peg test and 25-foot walk (HR 2.33 and 5.35, *p* = 0.036 and 0.003, respectively) [28]. In the EXPAND and INFORMS studies of siponimod in SPMS, and fingolimod in PPMS, respectively, a baseline bNFL > 30 pg/ml was associated with significantly greater confirmed disability progression (HR 1.32, *p* = 0.006 in SPMS; HR 1.49, *p* = 0.027 in PPMS) [42].

#### NFL and future MRI biomarkers of progression

Both baseline cNFL and bNFL have consistently been associated with future brain or spinal cord atrophy. In the EXPAND and INFORMS studies, baseline bNFL was grouped into low (< 30 pg/ml), medium (30–60 pg/ml) or high (> 60 pg/ml), and the high bNFL group experienced more than double the rate of brain atrophy at 24 months compared to low bNFL (*p* < 0.001, both studies) [42]. Similarly, in the ASCEND study of natalizumab in SPMS, higher baseline bNFL was associated with greater 96 week brain atrophy (*p* < 0.0001) [47], a finding replicated with cNFL in a smaller cohort from the MS-SMART study (also SPMS, *p* = 0.02) [65]. In a mixed PMS observational cohort, patients whose baseline bNFL was above the 99th percentile of a control cohort experienced greater brain and spinal cord atrophy at 2- and 5-year follow-ups [5].

#### NFL as a biomarker of treatment effect

Multiple studies have assessed cNFL as a biomarker of treatment effect in open-label studies in PMS. Significant reductions were demonstrated with natalizumab, rituximab or mitoxantrone, and in a mixed cohort starting various first- or second-line DMTs [3, 43, 60]. Case reports have reported similar findings with subcutaneous cladribine [66]. No treatment effect on cNFL was seen with monthly methylprednisolone, intrathecal mesenchymal stem cells, intrathecal and intravenous rituximab, intraventricular rituximab, dimethyl fumarate and intrathecal methotrexate [67–72].

In randomised, placebo controlled trials in PMS, a significant treatment effect upon bNFL has been shown with fingolimod, natalizumab, siponimod and ocrelizumab [28, 42, 47, 73]. With natalizumab, siponimod and ocrelizumab, the treatment effect is more marked in PMS with evidence of recent inflammatory activity at baseline (either relapses or GAD + lesions) compared to those without recent inflammatory activity. For natalizumab and siponimod, subgroups of patients without recent inflammatory activity still demonstrated a significant treatment response on bNFL [47, 73].

In contrast, a randomised, placebo controlled trial of ibudilast in PMS did not show a treatment effect upon bNFL, and in an open-label study of high-dose biotin [50], bNFL was not reduced following 2 years of treatment [48, 50].

Key results for NFL in PMS are summarised in Table 1.

**Table 1:** Key findings for neurofilament light in progressive multiple sclerosis—all eligible studies

	Supports	Against
NFL is higher in PMS compared to RRMS—all	Nine Studies, *n* = 609 RRMS vs. 312 PMS	14 studies, *n* = 1811 RRMS vs. 912 PMS
Difference in NFL between RR and PMS are lost if recent activity is excluded/covariates controlled for	Six studies, *n* = 610 RRMS vs. 298 PMS	–
NFL is associated with inflammatory activity	Nine studies, *n* = 3171	Three studies, *n* = 148
NFL is associated with current disability—EDSS	Six studies, *n* = 2036	11 studies, *n* = 476
NFL is associated with current disability—MSFC	One study, *n* = 744	–
NFL is associated with future disability worsening—EDSS	Three studies, *n* = 1881	Two studies, *n* = 587
NFL is associated with future brain atrophy	Four studies, *n* = 1680	–
Licenced disease-modifying therapies for RRMS show a treatment effect upon NFL in PMS—open label	Four studies, *n* = 111	One study, *n* = 16
Immunosuppressive disease-modifying therapies show a treatment effect upon NFL in PMS—RCTs	Four studies, *n* = 3090	–
Purportedly neuroprotective disease-modifying therapies show a treatment effect upon NFL—open label or RCT	–	Two studies, *n* = 320 (60 open label, 255 RCT)

Inflammatory activity was defined as either recent relapses, T1 GAD-enhancing lesions or new/enlarging T2 lesions

*NFL* neurofilament light, *RRMS* relapsing–remitting multiple sclerosis, *PMS* progressive multiple sclerosis, *EDSS* expanded disability status scale, *MSFC* multiple sclerosis functional composite, *RCT* randomised controlled trial

### Neurofilament heavy

#### NFH—associations with disease course

As for NFL, there is heterogeneity in reports comparing NFH in PMS and RR patients. Some studies have reported c/bNFH to be higher or increase more rapidly in PMS compared to RRMS or clinically isolated syndromes (CIS) [26, 74–78]; whilst others have found no difference [79–82].

#### NFH and current disability

Measuring disability by EDSS, 25FW, 9HPT, MSSS or ambulatory index, both cNFH and bNFH have been associated with current disability in PMS [54, 65, 76, 78, 83]. Other studies have found no association with EDSS [54, 79]. bNFH has also been associated with cognitive performance on the Paced Auditory Serial Addition Test (PASAT) [83].

#### NFH and MRI biomarkers

No data were found to support associations between NFH and MRI inflammatory activity. bNFH has, however, been associated with lower magnetisation transfer ratios and greater central cerebral volume loss, but not T2 lesion volume [83].

#### NFH and future disability

One study of cNFH has shown associations with future disability; whilst another associated a high blood–CSF NFH ratio with disability progression [65, 84]. Others have reported no such association [54, 83]. One study reported an association between baseline cNFH and subsequent whole-brain atrophy over 2 years [65].

#### NFH as a biomarker of treatment effect

Few studies have reported analyses of treatment effects using NFH. One very short study reported no change in cNFH over 8 days following treatment with intrathecal triamcinolone [82]. In an open-label study of HSCT, bNFH was significantly increased 1 month after HSCT, remaining elevated for 1 year. The increase was greater than that seen in haematology patients undergoing HSCT and untreated SPMS controls, suggesting possible vulnerability to chemotherapy-induced neurotoxicity in PMS [85]. In a randomised, placebo controlled trial of lamotrigine in SPMS, no treatment effect on bNFH was seen in the intention to treat population. Treatment compliance rates in this trial, however, were low, and on a secondary analysis of treatment-compliant participants, bNFH was reduced in those taking lamotrigine [83].

Key findings for NFH in PMS are summarised in Table 2.

**Table 2:** Key findings for neurofilament heavy in progressive multiple sclerosis—all eligible studies

Summary of key findings—NFH		
	Supports	Against
NFH is higher in PMS compared to RRMS—all	Three studies, 116 RRMS vs. 88 PMS	Four studies, 130 RRMS vs. 85 PMS
NFH is associated with current EDSS	Three studies, *n* = 217	Two studies, *n* = 79
NFH is associated with future disability	One study, *n* = 70	One study, *n* = 31

*NFH* neurofilament heavy, *RRMS* relapsing–remitting multiple sclerosis, *PMS* progressive multiple sclerosis, *EDSS* expanded disability status scale

### Summary of studies at low risk of bias included following QUADAS-2 review

Following QUADAS-2 scoring, 17 studies on NFL and three on NFH were found to be at low risk of bias. These consisted of larger cohort studies and randomised controlled trials, and are summarised in Table 3.

**Table 3:** Key findings from studies at low risk of bias on neurofilament light or neurofilament heavy in progressive multiple sclerosis

Study question	NFL or NFH	Studies	*n*	Reported results
Association with disease course	NFL	2	1757	bNFL is higher in SPMS compared to PPMS
		2	115	After controlling for significant covariates, bNFL not higher in PMS than RRMS
Association with current disability	NFL	4	1874	bNFL is associated with current EDSS
		6	1143	NFL is not associated with current EDSS (bNFL = 900, cNFL = 243)
		1	744	bNFL is associated with current 25FW and 9HPT
	NFH	2	190	NFH is associated with current or future EDSS (bNFH, *n* = 120, cNFH, *n* = 70)
	NFH	1	48	cNFH is not associated with EDSS
Association with current inflammatory activity	NFL	10	3533	NFL is associated with inflammatory activity. cNFL, *n* = 176; bNFL, *n* = 3357)
		2	133	bNFL is not associated with inflammatory activity
Association with future disability worsening	NFL	2	1757	bNFL is associated with increased risk of EDSS progression
		3	1330	NFL is not associated with increased risk of EDSS progression (bNFL, *n* = 1260; cNFL, *n* = 70)
		1	516	bNFL is associated with worsening 25FW and 9HPT
Association with future brain atrophy	NFL	5	2337	Baseline NFL is associated with future brain atrophy (bNFL, *n* = 2267; cNFL, *n* = 70
		1	68	Baseline bNFL is associated with future spinal atrophy
	NFH	1	70	Baseline cNFH is associated with future brain atrophy
Treatment effect	NFL	4	3020	bNFL is reduced by immunosuppressive disease-modifying treatment
		1	255	bNFL is not reduced by ibudilast
	NFH	1	120	bNFH is not reduced by lamotrigine in the ITT population, but is reduced in per-protocol compliant participants

Inflammatory activity was defined as either recent relapses, T1 GAD-enhancing lesions or new/enlarging T2 lesions

*bNFL* blood neurofilament light, *cNFL* CSF neurofilament light, *bNFH* blood neurofilament heavy, *cNFH* CSF neurofilament heavy, *RRMS* relapsing–remitting multiple sclerosis, *PMS* progressive multiple sclerosis, *EDSS* expanded disability status scale, *25FW* timed 25-foot walk, *9HPT* 9-hole peg test, *ITT* intension to treat ANALYSIS

The conclusions were broadly in line with those of all eligible studies. For NFL, the most consistent findings were found for associations with recent disease activity and future brain atrophy, and for the ability of immunosuppressive disease-modifying therapies to show a treatment effect upon blood NFL [3, 5, 28, 43–48, 50, 65, 73]. Associations between NFL and current or future disability were less consistent, and in the single positive phase 2 randomised controlled trial of a purportedly neuroprotective therapy, there was no treatment effect upon NFL [4, 10, 28, 35, 43, 44, 47, 48, 56, 58, 73]. Limited conclusions can be drawn from the few high-quality studies on NFH [65, 79, 83].

## Discussion

### Neurofilament light

The heterogeneity in reported data comparing NFL between patients with PMS and RRMS appears to be explained by associations with other covariates. Studies reporting higher NFL in RRMS compared to PMS often included a large proportion of RRMS patients during relapses, and in studies reporting higher NFL in PMS compared to RR, the PMS patients were older and a smaller proportion on DMT [4, 41]. The loss of significance between PMS and RRMS when either patients during relapse are excluded, or when multivariate analyses are undertaken including age, EDSS, recent relapses and DMT treatment status as covariates supports this [4, 5, 41].

The most consistent finding in the literature is the association between NFL and inflammatory disease activity in PMS. This replicates findings previously reported in the RRMS population. Whilst studies have also reported associations with cross-sectional clinical measures of disability, these results are less consistent. Associations with longitudinal disability progression are evident in larger cohorts, and are consistently demonstrated with MRI biomarkers of disability progression, such as brain and spinal cord atrophy.

The association of NFL with signs of active inflammation in PMS is supported by data on the ability of immunosuppressive DMTs to suppress NFL. Second line DMTs have consistently shown a treatment effect on NFL in PMS open-label studies. Whilst open-label studies are susceptible to bias and regression to the mean, such data have now been replicated in randomised controlled trials. Such findings, which have previously been demonstrated for RRMS, suggest that serial bNFL monitoring may be useful in the clinical monitoring of PMS as well as RRMS. As pwPMS tend to be older than pwRRMS, however, there is likely to be reduced signal-to-noise over the background increases in bNFL seen with aging [86], and vigilance will be necessary to exclude alternative sources of raised bNFL that are more prevalent in older PMS patients, such as peripheral neuropathy.

Studies which failed to show a treatment effect upon NFL included either unestablished treatments (mesenchymal stem cells, monthly methylprednisolone, intrathecal rituximab or methotrexate), small studies of first line DMTs (dimethyl fumarate, *n* = 16), or treatments with a purportedly neuroprotective, rather than immunosuppressive, mechanism of action. This latter group, based upon data from the phase 2 RCT of ibudilast and an open-label study of high-dose biotin, again supports the notion of NFL primarily being a marker of neuroinflammation, rather than neurodegeneration, in multiple sclerosis. This is further supported by the results of an RCT using oxcarbazepine, another purportedly neuroprotective treatment, as an add-on therapy in RRMS. Oxcarbazepine did not reduce the primary outcome of CSF NFL, but did slow the rate of disability progression on EDSS [87]. This further questions the utility of NFL as a marker of neuroprotection in multiple sclerosis.

Alternative explanations for the lack of treatment effect on NFL seen with purportedly neuroprotective treatments include the possibility that, compared to immunosuppressive treatments, they are not efficacious enough to demonstrate reductions in NFL, or that their efficacy involves mechanism that do not prevent NFL release. Indeed, in the follow-up phase 3 study, high-dose biotin did not meet its primary or secondary outcomes [88], and the efficacy of ibudilast has yet to be confirmed in a phase 3 trial. The association of NFL with imaging measures of neurodegeneration in non-inflammatory neurodegenerative dementias [89] and the normalised CSF NFL levels seen in response to treatment in spinal muscular atrophy [90] supports the utility of NFL as a biomarker of non-inflammatory neurodegeneration in other neurological conditions. More longitudinal data on NFL from trials of efficacious neuroprotective treatments are, therefore, required before firm conclusions can be reached. There is insufficient evidence at present to support the use of neurofilaments as primary outcome measures in phase 2 trials of *neuroprotective* therapies in progressive multiple sclerosis. Such trials are, therefore, likely to retain primary outcomes based upon measures of brain atrophy. The association of NFL with inflammatory disease activity and future brain atrophy, however, means NFL may help to identify patients with progressive multiple sclerosis who would benefit from combination therapies including both immunosuppressive and neuroprotective treatment strategies, or to assist in selecting patients for clinical trials who are likely to experience future accelerated brain atrophy, improving trial power.

### Neurofilament heavy

Less data were available on NFH compared to NFL. Multiple studies have reported associations with various measures of current and future disability, as well as MRI markers of future disease progression, but multiple negative results are also reported. Focusing on EDSS, four studies report cross-sectional or longitudinal association; whilst, three found no such associations. Due to heterogeneity in the literature, these findings, therefore, require further confirmation. Given the negative results from studies assessing the treatment effect of reportedly neuroprotective therapies on NFL, the per-protocol analysis finding of reduced bNFH in patients compliant with lamotrigine treatment is of interest. Lamotrigine is not immunosuppressive, and was investigated as a potential neuroprotective therapy. These data, however, must be treated with caution, as the lamotrigine compliant population consisted of only 50% of the intention to treat group [83]. Similar findings were found in a randomised controlled trial of phenytoin in optic neuritis, with a significant reduction in bNFH at 3 months in the phenytoin-treated group compared to controls [91]. The potential of NFH as a marker of neuroprotective treatment response, therefore, warrants further study. Caution is required with bNFH, however, as two studies have failed to show an association between cNFH and bNFH in PMS [80, 84]. Further research using current assay protocols is, therefore, required to confirm an association between bNFH, cNFH and other biomarkers of PMS pathology.

## Limitations

Limitations of this study include incomplete data availability and heterogeneity in the data assessed (for example, CSF and blood neurofilaments, different assays used for neurofilament quantification). A meta-analysis was, therefore, not undertaken, restricting the summary to a qualitative assessment of the literature.

## Conclusions

NFL has shown consistent utility as a biomarker of active neuroinflammation, future brain atrophy and immunosuppressive treatment response in PMS at a *group* level, and shows promising results as a disease intensity marker in non-inflammatory neurodegenerative diseases. Its performance as a biomarker of neurodegenerative pathology or neuroprotective treatment response in PMS is, however, uncertain and requires further research. The literature on NFH in PMS is smaller and less consistent, and whilst one study has suggested its utility as a potential biomarker of neuroprotection, this requires further confirmation.

## Availability of data and material (data transparency)

Online supplementary material included.

## Electronic supplementary material

Below is the link to the electronic supplementary material.

Supplementary file1 (XLSX 67 kb)
